# Exploring the lived experience of mental health and coping during unemployment

**DOI:** 10.1186/s12889-022-14858-3

**Published:** 2022-12-28

**Authors:** Andrew F. Arena, Marnie Harris, Sophia Mobbs, Alexandra Nicolopoulos, Samuel B. Harvey, Mark Deady

**Affiliations:** Black Dog Institute, Faculty of Medicine and Health, University of New South Wales, Hospital Rd, Randwick, NSW 2031 Sydney, Australia

**Keywords:** Unemployment, Mental health, Coping, Lived experience, Phenomenology, Thematic analysis

## Abstract

**Background:**

Unemployment is known to involve various psychosocial challenges that can negatively impact mental health. However, the intricacies of how individuals experience these challenges and strive to cope within the context of varied sociocultural and individuating factors, remain comparatively understudied. The present qualitative study used an interpretative phenomenological approach to explore the lived experiences of mental health and coping during unemployment.

**Methods:**

Fifteen Australian adults who had recently experienced unemployment (for ≥3 months in the last 2 years), despite being available for and able to work, participated in semi-structured interviews from August to September 2021. Maximum variation sampling ensured participants represented diverse sociodemographic backgrounds. Interviews were audio-recorded, transcribed verbatim and analysed using reflexive thematic analysis within NVivo12 software.

**Results:**

Four major themes were identified: 1) disrupted identity and direction in life; 2) navigating conflicting views of contribution and progress; 3) knowing how to cope is not enough; and 4) unemployment as a catalyst for new understandings. Unemployment disrupted participants’ sense of purpose, identity and visions for the future. It signified a perceived failure to meet societal standards of value based upon the economic functions of work, which participants struggled to reconcile with their own priorities for work that satisfied psychosocial needs. Participants were aware of effective coping strategies, although these had mixed positive and negative effects on mental health, or were difficult to mobilise during unemployment. The COVID-19 pandemic, while normalising unemployment to some degree, exacerbated future uncertainty and prevented engagement with known coping strategies (e.g., social interaction). However, unemployment could also instigate growth through re-defining markers of achievement, re-aligning goals with one’s core values, and developing greater compassion.

**Conclusions:**

Experiences of mental health and coping during unemployment share complex relationships both with each other and with broader personal and sociocultural contexts. Service providers may better meet the mental health needs of those experiencing unemployment by balancing the economic and psychosocial functions of work, understanding that coping is a wholistic issue that goes beyond knowledge of effective strategies, and being aware of the opportunities for self-development that unemployment can create.

**Supplementary Information:**

The online version contains supplementary material available at 10.1186/s12889-022-14858-3.

## Background

It is widely established that experiencing unemployment is associated with poor mental health and well-being outcomes [1, 2], with job loss argued to be amongst the most stressful life events [3–5]. There is evidence that this relationship is bi-directional in nature, whereby poor mental health both constitutes a common outcome of unemployment in addition to increasing the risk of unemployment [6, 7]. This has the potential to maintain a cycle of socioeconomic disadvantage and suggests a need to consider experiences of unemployment and mental health as closely intertwined. Unemployment can therefore be seen as both an economic and public health concern, further highlighted by its association with increased rates of suicide [8] and all-cause mortality [9].

During major economic crises like that brought about by the COVID-19 pandemic, unemployment and mental health become increasingly prominent public health issues [10–12]. In the early stages of the COVID-19 pandemic in Australia, unemployment rates rose rapidly by 40% from March to July 2020 [13], and rates of severe psychological distress amongst those who lost their job were estimated to be up to 8.4 times higher than for those whose work was unaffected, depending on levels of financial resources and social interaction [14]. Mass unemployment can result in deeply existential experiences of loss and anxiety, brought about by broad threats to fundamental needs for survival, social connection and self-determination [15]. The potential for cascading or delayed mental health impacts of unemployment and economic hardship [16–19] suggests that the impact of economic crises continue to be a public health concern long after the initial event. Other socioeconomic factors, including the nature of welfare systems and active labour market programs available to people during economic hardship, can also impact mental health [20, 21]. Taken together this highlights the need for research to consider the experience of unemployment as uniquely situated within, and shaped by, broad socioeconomic and sociocultural contexts.

Specific individual-level pressures pertaining to the experience of unemployment have also been shown to contribute to poor mental health and well-being. Unsurprisingly, both objective and perceived financial strain have commonly been identified as among the most important predictors of poor mental health and well-being during unemployment, which may be partly explained by decreased access to coping resources [2, 22, 23]. The job-seeking process, typically involving repeated rejections, is also widely acknowledged to be a stressful experience associated with negative psychological outcomes [2, 24, 25]. While these features of the unemployment experience are often detrimental, those who are less identified with their work role and who have greater coping resources (including more positive core self-evaluations, social support, and structured use of time) tend to fare better during unemployment [2]. Demographic features commonly associated with a stronger negative relationship between unemployment and mental health or well-being include male gender and longer duration of unemployment [1]. The moderating role of age is complex, with meta-analyses finding partial evidence of a U-shaped relationship (unemployment predicting worse mental health outcomes for younger and older age groups [1]) or evidence of a stronger relationship for school leavers compared to adults [2]. Ultimately, the heterogeneity in the experience of mental health problems during unemployment suggests that to fully grasp this phenomenon, it is imperative to consider individuals’ unique experiences as embedded within their personal and situational contexts.

By going beyond a description of mental health symptoms and their correlates, qualitative research in this field can offer a depth of understanding regarding the mental health issues experienced during unemployment, the efforts individuals undertake to cope and how contextual factors influence and intersect with these experiences. Such knowledge could contribute to the design, tailoring and improvement of mental health prevention and promotion strategies for unemployed people, which has the potential to enhance their effectiveness in terms of mental health, well-being, and re-employment outcomes [26].

To date, much of the qualitative literature in this area has focused on how unemployment is experienced by those with severe mental health issues [27–31] and other specific subgroups (e.g. age groups, genders or durations of unemployment [32–37]). Others have focused on the impact of particular contextual factors [38], collected data using open-ended questions [39], or have taken a deductive approach to explore specific theoretical concepts [40–43]. Substantial knowledge has been gained from this research, in particular around describing the types of psychological challenges faced during unemployment (e.g., threats to one’s sense of purpose and identity) and identifying commonly used coping strategies (e.g., structuring one’s time and seeking social support). However, relatively few studies have examined the lived experience of unemployment in combination with varied contextual factors in the general population, or taken an interpretive phenomenological approach to understanding these experiences. Such an approach offers considerable value here, by prioritising the interpreted meaning of a phenomenon as it emerges within particular contexts (like the COVID-19 pandemic), and enabling an in-depth investigation beyond simply what the commonalities of an experience are, to how they are experienced [44, 45].

The present study therefore aims to build on the existing literature by taking an inductive, interpretive phenomenological approach to understanding the lived experience of mental health and coping efforts during unemployment, whilst also exploring any distinct experiences that arise in the context of broad sociocultural and relevant individuating factors (gender, age, and duration of unemployment). Acknowledging that unemployment is also contextualised by temporal-spatial and cultural factors, the present study contributes to the recent literature by examining the experiences of an Australian sample in the post-COVID-19 era, and investigating the interplay between mental health, coping and one’s wider sociocultural context.

## Method

The current study’s theoretical framework and philosophical positioning drew on the interpretive, Heideggerian phenomenological tradition [44–46]. This approach holds that ‘being’ only occurs in a particular context (dasein), and thus any experience of a phenomenon is inextricably situated within a sociocultural and geographic environment. Furthermore, the meaning of experiences is held to be constructed and interpreted through an embodied, subjective lens, both for the individual experiencing the phenomenon and the researcher studying these experiences. While this approach emphasizes the understanding of an individual’s lived experience from their own situated point of view as it naturally unfolds in their life, knowledge of the phenomenon under study is co-constructed by both the individual and the researcher. Thus, the researcher’s personal history and lived experience cannot be bracketed when interpreting data, and there is rather a preference for reflexively acknowledging and remaining aware of one’s personal perspective and influence throughout the qualitative analysis process.

### Sample and recruitment

All study methods were approved by the University of New South Wales Human Research Ethics Committee (HC210358). In line with recommendations for phenomenological approaches, purposive sampling was used to target participants with a rich understanding of the phenomenon of interest, and ensure a necessary degree of homogeneity in the phenomenon being studied [44, 45, 47]. To this end, eligible participants were (1) Australian residents aged 18–65 years, (2) fluent in English, (3) had experienced unemployment for at least 3 months in the last 2 years, and (4) during their period of unemployment, were both available for and able to work (e.g., did not have full-time study or caring responsibilities, or did not have injuries or disabilities precluding work). Simultaneously, the study aimed to capture a breadth of unemployment experiences, and thus employed maximum variation sampling [45] by iteratively adjusting the recruitment strategy to ensure variation in age, gender, duration of unemployment, and education level.

A sample of 15 participants was targeted, based upon both the anticipated information power with the present research aims and methodologies [48] and approximate norms within phenomenological approaches [45, 49]. Data saturation was not used to justify the sample size due to concerns around its appropriateness for an interpretive, reflexive thematic analysis, although there was a provision to recruit further participants into the study should the data be insufficiently rich and complex to address our aims [50].

The study was promoted on the website and social media pages of a well-known Sydney-based mental health research institute. Prospective participants were linked to a webpage where they received information on the study (e.g., aims and requirements for involvement), could register their interest, and answer a brief screening questionnaire. Eligibility was confirmed with all participants individually before inviting them into the study. All participants signed and e-mailed informed consent forms to the researchers prior to their interview. Of the interested individuals who were approached for participation, four were ineligible and seven were unresponsive. All participants were reimbursed for their time with an e-gift card worth AUD$60.

### Data collection

Semi-structured interviews were conducted via Zoom videoconferencing from August to September 2021. In line with recommendations [45], interviews were scheduled for approximately 1 h to ensure sufficient time to cover a wide breadth of potential topics, although participants were able to take as much time as they needed to fully explore their experiences; range = 45–76 minutes, mean = 63.32, SD = 8.93. During this time, Australia was considerably impacted by social distancing measures due to COVID-19 outbreaks. Although the national unemployment rate during this time was comparatively low (4.5–4.7%) [51], Australia’s most populous states were in the midst of their longest and most stringent restrictions with an uncertain path to recovery, which substantially limited economic activity [52]. After initially calibrating interview style through mock interviews, an approximately equal number of interviews were conducted one-on-one with participants by AA or SM. Both interviewers met regularly to discuss how the interview process was unfolding and reflect on how to optimally engage with participants.

Interviewers began by introducing themselves, the goals, expectations and safety protocols of the research, and getting to know the participant (including their demographic background). To allow participants to guide the direction and focus of the interview based on what was important to their own unique experiences, the opening interview question was intentionally broad, and additional guiding questions and prompts were flexibly used to encourage a deep, multifaceted conceptualization of their mental health and coping (see Table 1).

**Table 1:** Interview topic guide

Opening question	
1. Tell me about your experience of unemployment—what was it like for you?	
Potential follow-up questions	
1. How would you describe your mental health typically? And how would you describe your mental health during unemployment? 2. Is there anything you did/do while unemployed to manage your own mental health? 3. While unemployed, was there any point where you sought out help for your mental health? If so, how did you go about doing that? Was it helpful? 4. How would you describe your social relationships during unemployment (E.g., partners, friends, past co-workers, social groups)? 5. How would you describe your involvement in society/the community during unemployment? 6. Have you had anyone treat or think of you differently while unemployed? 7. Have you thought or felt differently about yourself while unemployed? 8. Is there something we haven’t asked you that you think might be valuable for us to know?	
General prompts	
1. Could you tell me a bit more about that experience? 2. Can you think of a concrete example of that? 3. Did that have an impact on your mental health/well-being/other areas of your life? 4. What do you think would have been needed for your mental health/well-being in that situation? 5. How does that compare to before/after unemployment?	

### Data analysis

Interviews were audio-recorded, transcribed verbatim and coded using NVivo12 software. Reflexive Thematic Analysis was used to analyse the data [53, 54], taking an inductive approach to theme generation, driven bottom-up by the expressed lived experiences of participants. Two researchers immersed themselves in all transcripts to ensure familiarity with the complete dataset, and both independently coded a subset of 4 transcripts (27%). After discussing and refining the approach to the coding, and any impressions of potential emerging themes in the data, one researcher coded the remaining transcripts. Both coders independently organized all codes into subthemes, allowing for latent underlying meanings and context to inform the interpretation of thematic content. To further increase trustworthiness, a third researcher reviewed the organization of 30% of these codes into subthemes, before all three researchers collaboratively refined the interpretive decisions made. The subthemes were then organized into higher-order themes by the two original coders, and refined in collaboration with a fourth researcher. Transcripts were then re-examined to probe for any experiences that were markedly contextualized by participants’ gender, age (grouped by tertiles) or duration of unemployment (short- and long-term defined as < or ≥ 12 months) [55].

### Researchers’ perspectives and reflexivity

Beginning prior to conducting the first interviews, both interviewers kept a reflexive journal and met regularly to document and discuss their personal and professional perspectives, and initial impressions of either the interview processes or potential thematic content. Coders also engaged in these reflexive processes, to ensure an open, honest and reflective approach to theme generation. Such methods are considered essential within Reflexive Thematic Analysis and phenomenological approaches, as they foster greater attunement to the data and a rich, authentic interpretive discourse around the phenomenon [45, 54]. All researchers shared a collective motivation to better understand and improve mental health, and to draw out potential applications and translatable implications of participants’ experiences. The researchers were partly interested in the project based on its potential to inform the development of digital technologies to support the well-being of this population, and this research interest was disclosed to all participants.

## Results

A total of 15 individuals participated, aged between 23 and 62 years (mean = 37.67, SD = 13.21; tertile categories 18–29, 30–44, and 45–65). The sample composition was 53% women (40% men, 7% non-binary), 40% long-term unemployed (≥ 1 year), 53% university educated, 66% Caucasian ethnicity (20% Asian, 7% Hispanic, 7% Middle-Eastern), and 53% currently unemployed. Participants came from diverse occupational backgrounds, with two participants each most recently working in retail, human resources, research, and mental health/disability fields, and one participant each within education, design, software engineering, fundraising, international development, construction and debt collection.

As a demonstration of the reflexive co-construction of meaning, the researchers entered interviews with an awareness of the negative attitudes that often exist between unemployed groups and government systems, which was documented early in our journaling during the interview stage; “Perhaps unsurprisingly … it seems we’re both coming across the government as a core source of stigma and feelings of disrespect”. After delving deeper into the coding of meaning ascribed to these experiences by participants, the underlying pressures being expressed in these sentiments came from many sources. Through analytic discussions, the researchers reviewed the transcripts and coding, interpreting these pressures as “more than just an issue of government attitudes, but is an issue with societal expectations and assumptions.” In this instance, these discussions in turn led to the refining of Theme 2 below, exemplifying the reflexive approach to theme generation.

Qualitative analysis of interviews identified four over-arching themes: (1) disrupted identity and direction in life; (2) navigating conflicting views of contribution and progress; (3) knowing how to cope is not enough; and (4) unemployment as a catalyst for new understandings. Some differences in the experiences of sub-groups were found and are noted where they occurred. Quotes are identified by participant ID (P), gender (F, M, NB), duration of unemployment (ST, LT), and age (18–65). See Additional file 1 for further quotes pertaining to each theme.

### Theme 1: disrupted identity and direction in life

Overall, participants described unemployment as a “really difficult” (P10_M_ST_45–65) experience, in which they had to deal with both job loss and a complex mix of consequent psychosocial stressors. Work provided participants with a sense of identity, purpose, and a way to structure their time; all of which were disrupted with job loss. Most participants described enduring days void of direction, purpose, and a sense of achievement. For some, days spent doing “nothing, really” (P03_F_LT_30–44) promoted depressive thinking and low mood, which fed back into a loss of motivation for purposeful activities: “...there was a very vague feeling of just … waffling through. With no mile markers for ‘I did a thing’” (P01_F_LT_30–44). Additionally, for participants who highly identified with work, job loss disrupted their identity and prompted them to question who they are: “I felt like I lost myself. Yeah, I had worked from a very young age” (P06_F_ST_18–29).

Job loss also elicited a sense of uncertainty about the future, both generally (“what am I going to do?”; P07_F_ST_45–65) and specifically relating to future employment. Participants aged 45–65 raised concerns that their skills and experience would not be seen as relevant in the current job market, which contributed to worry about future employment and financial security. COVID-19 exacerbated these uncertainties by disrupting participants’ taken-for-granted understandings of life, the future and the prospect of finding employment again.

“When we kind of officially went into lockdown, every job posting or every interview I had locked in just … went. So I started to feel quite hopeless. I had a lot of feelings of depression, it was really hard for me to get out of bed some days because I just felt like I really had no purpose” (P06_F_ST_18–29).

This extended to uncertainty about being able to stay in the country for one participant on a working visa. Ultimately, the uncertainty and loss of control over one’s life—particularly within the context of the COVID-19 pandemic—was a chronic source of stress and caused feelings of dejection.

### Theme 2: navigating conflicting views of contribution and progress

Participants described a pressure from others to work and be financially independent, which were assumed to be requirements of a productive member of society making a meaningful contribution. Everyday comments and questions, such as “what do you do for work?” and “aren’t you working yet?” were received as pejoratively implying that all individuals should be in paid employment, and that spending any period of time without it was unacceptable. This assumption was reinforced in interactions with friends, family and the welfare system, whereby participants felt pressured to apply for or accept any job, even those misaligned with their skills, interests and career goals. These interactions (particularly prominent within short-term unemployment experiences) also portrayed work solely as a means of earning money and economically contributing to society, marginalising its psychosocial functions (e.g., providing a sense of purpose) which were valuable to participants.

“[Welfare providers] have no nuance in the system, they have no understanding that people aren’t... just wasting their time in exchange for income. The point of work is to give you meaning, and to actually be someone …” (P13_M_LT_30–44).

This pressure, coupled with participants’ exposure to, or awareness of stigma surrounding unemployed people (e.g., as “dole bludgers”, “leeching off the taxpayer”) conveyed the impression that making progress and having value in society means being economically active, leaving little room for other means of progress or contribution outside of paid employment.

“The typical thing is, when you meet someone new is ‘What do you do?’ Like, how do I define you in society and set you on a, you know, hierarchy of importance or value? So then when I say I’m not working, they’re like, ‘Oh, oh okay’” (P03_F_LT_30–44).

“I don’t feel like I’ve got a connection [to society] at all. I feel like I’m worth nothing because I don’t have a job. And people have jobs. That’s what people do, you know?” (P04_F_LT_45–65).

The prevailing attitude that individuals should, at all times, be financially contributing to society was internalised by participants, evidenced in their comments about feeling the need to look for work or contribute as “the right thing” to do (P02_M_LT_45–65), and guilt for not doing so. Despite acknowledging that COVID-19 had made it quite difficult for people to find work, any normalisation of unemployment in this context was overridden by prevailing internalised pressures to be employed.

“There’s a part of me that knows ‘Hey, look, we’re in lockdown at the moment, and getting a job is probably not too easy, or not too wise … ’ But there’s still this sense of me that’s like, ‘You should go out and get a job, like no matter what’” (P14_NB_LT_18–29).

Unemployment signified a perceived personal failure to meet societal expectations (both external and internalized), and particularly for those aged 18–29, a failure to meet expectations of making sufficient life and career progress compared to others: “These are what expectations I’d set on myself, and other people had set on me to achieve at this point in time. And I’m very far away from that” (P11_M_ST_18–29). These pressures decreased participants’ self-worth, and particularly for those aged 30–65, contributed to feelings of shame and embarrassment: “I was embarrassed to be me. And I guess part of it was, you know, just feeling not good enough.”

“… one of the biggest issues is feeling like I’m not doing enough for myself or my husband, or anyone” (P14_NB_LT_18–29).

During unemployment, participants were forced to grapple with the conflict between these sociocultural views and their own personal views of work, contribution, and progress. Participants consistently expressed a desire for work that was aligned with their qualifications, skills and interests, which would give them a sense of purpose, stimulation and help them to progress broader career or life goals (as opposed to simply progressing out of unemployment). This was exemplified in participants’ desire to “get back to” where they were in their careers before unemployment, and for those who became re-employed, frustration or disappointment at working in jobs they were overqualified for. There was discord between participants’ desire for personal progress and fulfilment through work, and others’ pressure to prioritise work’s financial functions, which was difficult for participants to reconcile and negatively impacted mental health. Additionally, striving to meet both societal and personal standards of progress added to the stress of job seeking. While participants also desired to contribute financially within their relationships and to society more broadly, this existed alongside their desires for meaningful work and progress, rather than being their sole concern.

“I don’t want to take a shitty low paid job just because someone says you should be working. I’m like, ‘No, I worked my butt off for 15 years to get where I was. I want to go back to that, because that’s what I want to pursue. I want my future to be in a certain direction. I want to be able to build a career and financial independence’” (P03_F_LT_30–44).

### Theme 3: knowing how to cope is not enough

Participants had an awareness of what they needed to do to cope, though during unemployment these means of coping had mixed positive and negative effects on mental health and well-being, or were difficult to mobilise.

#### Theme 3.1: known coping strategies can help but also hinder mental health

Experiencing unemployment was financially stressful, and to support their mental health, participants often sought financial support from loved ones or the welfare system. While both supports helped to alleviate financial strain, they did not entirely relieve stress and contributed to feelings of shame and diminished self-worth. Relying on financial support made participants feel dependant on others and burdensome: “… one of the central things is embarrassment. A feeling of … needing to get a loan and not being able to stand up on my own feet” (P05_M_ST_30–44). Welfare payments were insufficient to support normal living expenses for some participants (covering “the essential things” only; P06_F_ST_18–29), who continued to experience financial stress. In addition, many participants described negative experiences accessing these payments, whereby participants’ unique circumstances and aspirations for work were disregarded, making them feel “like a number” (P06_F_ST_18–29). Schemes provided by the system to help people find employment (e.g., working for payments) and unrealistic mutual obligation requirements were considered unhelpful for securing employment and contributed additional pressure and stress to the job-seeking process. These pressures, in addition to shame and stigma, contributed to a reluctance to seek financial support amongst short-term unemployed participants.

“[mandated employment services meetings are] just a stress. Like, I’m gonna have to go talk to someone that has pretty much no emotion, and treats you like you’re not good enough and you haven’t done enough. Instead of getting mental health support, you’re getting actual blame and guilt. … that’s why I chose not to continue [receiving welfare payments] despite the financial stress, because it just wasn’t worth talking to someone that doesn’t care at all.” (P05__M_ST_30–44).

Job-seeking was a coping strategy used to alleviate financial stress and regain a sense of purpose, achievement and identity. However, the process of job-seeking was often demoralising. Receiving rejections or not hearing back from employers represented a “constant cycle of hope and disappointment” (P13_M_LT_30–44), which ultimately decreased participants’ self-worth and confidence in their ability to find or keep work in the future. Increased unemployment rates and a paucity of jobs available during the COVID-19 pandemic meant the odds of finding work were low and rejection was common and often poorly communicated, exacerbating these feelings. In recognising these negative impacts that job-seeking could have on mental health, some participants set clear boundaries around job-seeking (e.g., taking time off after rejection, setting specific hours for job-seeking), although sometimes experienced guilt for doing so.

“… they don’t get back to you … The ones that did get back to me, they sent me a lower pay scale and things like that … It was demoralising. It just made me feel like, maybe I can’t do my job. Maybe I’m not good enough” (P12_F_ST_45–65).

Furthermore, while successfully securing a job after a period of unemployment eased financial strain, participants had difficulty re-establishing a routine and experienced anxiety surrounding their capabilities and the possibility of losing their job again.

“I just felt … less capable of doing it. Because I wasn’t doing it for so long … Which probably leads into the anxiety I had when I first started this role around … ‘Am I out of the game?’ … Coupled with ‘is this going to happen again to me?’” (P06_F_ST_18–29).

Participants also experienced significant social isolation during unemployment, and seeking social connection was recognised as an important means of coping. Interacting with others who were empathetic and understanding of participants’ situations, and who did not pressure them to find work, provided a sense of quality connection otherwise lost during unemployment and helped to bolster self-esteem: “[my partner was] very supportive in hyping up the small wins … Even if it was just, like, I applied for one job that day...” (P11_M_ST_18–29). Connecting with others that were similarly experiencing unemployment or mental health issues normalised and validated participants’ experiences, helping to combat their isolation. Participants acknowledged that these challenges were common during the COVID-19 pandemic, which provided a sense of solidarity: “… an element of surviving it is just knowing that I’m not alone in it. That this is actually happening to lots and lots of people” (P03_F_LT_30–44).

However, interacting with people who lacked an understanding of participants’ situations or were judgemental could exacerbate isolation and be detrimental to mental health. Even when well-intentioned, pressure from others to seek work that was misaligned with participants’ skills and aspirations increased social disconnection, as it signalled that they were being misunderstood, undervalued or ignored. Some participants (particularly those who had experienced long-term unemployment) felt judged by others for being unemployed, which again was isolating and decreased self-worth: “When you say [to other people] you’re unemployed, it’s kind of like [groans and rolls eyes] ‘You’re unemployed.’ But then also I feel it myself, you know, [groans and rolls eyes] ‘I’m unemployed.’” (P04_F_LT_45–65).

#### Theme 3.2: effective coping strategies are known, but challenging to mobilize

Participants described various strategies that effectively facilitated coping and improved mental health and well-being during unemployment, including reflective activities (e.g., mindfulness and journaling), exercise and eating well, identifying and challenging maladaptive thoughts, taking time to feel and process job loss, positive self-talk and positive reframing, and finding ways to restore routine, a sense of purpose and productivity. Despite their awareness of these strategies, participants often struggled to mobilise them during unemployment. A lack of motivation or energy prevented participants’ initial or sustained engagement in activities known to facilitate coping. A loss of mental clarity or capacity during unemployment, sometimes due to acute stress and anxiety, made reflective activities difficult or impossible: “when I was really anxious, or when my mental health was really struggling, I couldn’t read … I’d read two words, and it would be like, ‘I don’t even know what I just read’” (P09_F_ST_18–29). For some, engagement with known coping strategies depended upon their mood; engagement was high when they were feeling good, but low when they were depressed or emotionally fatigued. Negative previous employment and job loss experiences (reported by female participants), or pre-existing mental health issues increased participants’ psychological strain and depleted their coping capacity. Participants also found positive self-talk difficult to maintain and some, over time, became self-critical.

“… exercise, psychologist, and then just changing habits. Making small adjustments to daily life and being consistent with it … but I struggle with that on a daily basis to be honest with you. Some days, I don’t want to do anything” (P10_M_ST_45–65).

“...for the first three months, I had the capacity to be kind to myself about it. To say, ‘be kinder. The world is completely turned upside down. It’s normal to feel the trauma.’ So that was okay, for three months or so … Then it became—I would get harder on myself” (P01_F_LT_30–44).

The financial strain of unemployment prevented some participants from socialising or accessing professional psychological support. Establishing quality social connection was hindered by participants’ intentional social withdrawal and reluctance to seek help due to low mood and consequent lack of motivation, shame and embarrassment about their situation, desire not to burden others with their problems, or a perception that others “might not understand, or they might not see it from my perspective” (P11_M_ST_18–29).

“[When I became unemployed] I think I withdrew from my life in general. I think I spent the next week in bed claiming I was feeling unwell, but it was more like, mentally I just couldn’t face … anyone or anything” (P08_F_ST_30–44).

COVID-19 and the ensuing lockdowns added to the difficulty of engaging with known coping strategies, by preventing travel, regular activities, and inflating the demand for psychological support making wait times exorbitant. Additionally, socialising with others in-person during the COVID-19 lockdowns was difficult or unachievable, and online modalities were “not quite the same” (P02_M_LT_45–65) in fostering a genuine sense of connection. Concerningly, for some the lockdowns facilitated their intentional withdrawal from others and everyday activities (“an excuse … to hide away”; P10_M_ST_45–65).

### Theme 4: unemployment as a catalyst for new understandings

Unemployment prompted participants to reassess their taken-for-granted assumptions about work, life, and the world, leading to new understandings that typically benefited mental health and coping. Through their experience of unemployment, participants developed new understandings of achievement and meaningful contribution; needs previously fulfilled through paid employment. Completing everyday tasks or maintaining healthy habits despite unemployment-related challenges were, amongst other things, adopted as new markers of achievement: “I feel like I started to realise the value in noticing the little things that you do each day when they’re the only things” (P09_F_ST_18–29). Participants also found new, non-financial ways to contribute, for example through volunteer work and providing practical support to others.

Unemployment caused participants to scrutinise their values and previous employment experiences, and for some male participants, raised broad existential questions about the meaning of work and life in general. Whilst difficult, these processes could lead to shifts toward more personally meaningful work or life values, priorities and goals: “… this experience is showing me that I need to make [my daughter] more of a priority … that’s definitely a positive that’s come out of this” (P08_F_ST_30–44). For some, seeing a stark incongruence between their careers and values prompted the decision to seek work that aligned with altruistic rather than financial motives, and that would allow for greater work-life balance, which was positive: “as hard as it’s been, it’s given me a chance to realign morals, values. Make sure I’m actually true to myself as well” (P10_M_ST_45–65). Occasionally, participants’ unemployment led to disillusionment with large organisations or capitalistic systems in general, particularly in light of the COVID-19 pandemic: “… the game is rigged against us, but I also have to be a part of it. So, it’s just like, why do I want to be a part of it if it’s so rigged?” (P11_M_ST_18–29).

Experiencing unemployment and the associated struggle to maintain mental health and well-being also prompted participants to reconsider their previously held views of unemployed people. Several participants reflected that being unemployed was harder than they’d anticipated—a “humbling experience” (P07_F_ST_45–65) that imparted a new sense of empathy and social justice for others struggling with unemployment or mental health challenges.

“And feeling like, maybe when I was young I might have had that dole bludger attitude too, just from the media. Not realising it would be me one day. Even, like, being in the Centrelink queue and thinking, ‘what the hell am I doing here? I’m not like these people.’ [Laughs] You know?” (P13_M_LT_30–44).

“I think the fact that I was resenting the people that could sit at home and get paid and do nothing sums it up. Like, I didn’t expect it to be so hard to not have something to wake up for.” (P09_F_ST_18–29).

## Discussion

This study took an interpretive phenomenological approach to explore how mental health and coping are experienced during unemployment, and how these experiences are situated within sociocultural or personal contexts. The lived experience of participants centred around four themes, which are ultimately characterised by a series of tensions between: one’s established life before unemployment versus their disrupted life during unemployment (Theme 1); societal versus personal views of contribution and progress (Theme 2); striving to cope versus challenges which impede coping (Theme 3); and one’s prior worldview versus newly established understandings (Theme 4). The four themes captured distinct experiential features, yet often impacted each other, which is perhaps unsurprising given that problems faced during unemployment have been understood to be multifaceted and intertwined [30, 42]. For instance, the social construction of unemployed people as not productively contributing to society expressed in Theme 2 made participants feel ashamed or embarrassed for being unemployed, contributing to participants’ reluctance to seek financial support and intentional social withdrawal (Theme 3). While some experiences of participants were notably contextualised by sociodemographic factors, these were largely outweighed by the impact of broader sociocultural and economic contexts. These findings contribute to a rich understanding of how societal pressures interact with mental health and coping during unemployment, what people know and struggle with in their journey toward successful coping, and how people can sometimes find value in their challenges and experience personal growth.

### Disrupted identity and direction in life

The broad disruption to life that adversely impacted the mental health of participants is unsurprising given what is generally observed in the literature on unemployment. According to the latent deprivation model, a sense of purpose and identity are two key psychosocial functions of work which are thwarted during unemployment [56, 57], having a considerable impact of the mental health of participants. Compared to their life before unemployment, participants felt directionless without work to help define themselves and their path forward in life. The loss of their structured sense of achievement and purpose fed into a cycle of melancholy and diminished capacity to complete daily tasks, hindering the ability to rebuild a sense of achievement and purpose. The findings generally align with the bi-directional relationship between mental health and unemployment found elsewhere [6, 7, 32]. The disruption caused by job loss had negative consequences for mental health, and the psychological hardship faced during unemployment often negatively impacted job-seeking and other forms of coping, threatening to maintain unemployment.

The disrupted sense of one’s future employability was widely prevalent across participants’ experiences, and greatly compounded by the loss of a predictable future caused by the COVID-19 pandemic. The older age of some participants further exacerbated these insecurities, as job loss caused them to negatively reassess the value of their skillset and identity as an employable person, which made future employment seem even more tenuous. The hampered construction of a meaningful identity and future were closely intertwined among participants, and both highly consequential to mental health.

### Navigating conflicting views of contribution and progress

Mental health was negatively impacted by the socially constructed meaning of a valuable, accepted member of society as someone who is, above all else, making an economic contribution. This builds on other qualitative findings that without work, people feel excluded from society [28, 32, 34]. It was not the implication that participants needed to find paid work that was harmful in itself (indeed, this was typically participants’ primary goal), but rather the sense of a prevailing societal attitude that portrayed anyone not in paid work for a period of time as deviant, even when this is outside of their control. In this context, a normal, common experience of job loss is interpreted as unacceptable, exacerbating perceptions of failure and loss of self-worth. The sense of failure and loss of self-worth appeared to be contextualised by the life stage of participants, whereby they perceived unemployment as inappropriate for their age group. For younger participants, direct upward social comparisons with peers, led them to feel they were not making sufficient progress toward the normative developmental task of establishing themselves in the work world. Older participants also interpreted unemployment as inappropriate for their life stage, although expressed greater shame and embarrassment around the feeling that they ‘should’ be working as an established, independent and productive member of society. Overall, the fact that these experiences emerged so strongly despite a degree of normalisation of unemployment during the COVID-19 pandemic, speaks to the pervasiveness of these social constructions and the depth to which they become internalised.

This view of work almost exclusively prioritises its manifest economic function to satisfy survival needs [57], which conflicts with core meanings that participants ascribed to work and the types of contributions and progress they considered valuable. Participants viewed work as a means of satisfying latent psychosocial functions, including the purposeful engagement of their skills and values in pursuit of higher-order goals [57]. This conflict was felt particularly strong during short-term unemployment as participants navigated an unfamiliar socioeconomic situation, and among other things, can contribute to feeling misunderstood, devalued and distrusting of others. It therefore comprises an important consideration for clinicians, employment services and welfare systems. If service providers working in this area are to concern themselves with the mental health and well-being of people experiencing unemployment, their focus cannot solely be on securing any job as quickly as possible. While this can help to resolve survival needs, it may not satisfy psychosocial needs that underpin mental health, which may in turn threaten the sustainability of the re-employment and decrease confidence in the relevant service.

### Knowing how to cope is not enough

Participants expressed an awareness of many useful ways of coping, although their effectiveness was thwarted by various negative experiences arising from their efforts, or by various barriers to engaging in these efforts at all. Sourcing financial assistance from welfare systems or through social connections was an important coping strategy to meet basic mental health needs while individuals were job-seeking or not yet able to seek jobs, yet this was met with challenges that simultaneously undermined mental health. Despite providing practical benefits, seeking financial assistance was a widely unpleasant and stressful experience, as it triggered negative self-evaluations of being a burden on others or on society that conflicted with a keen desire to be self-sufficient. Individuals’ self-worth was considerably derived from their independence, competencies, and productivity, contrasting against the prevailing denigratory stereotypes of unemployed people as disingenuous and ‘leeching off’ taxpayers during unemployment. The act of engaging with welfare systems therefore already represented a threat to participants’ self-worth, which meant that common dehumanising and adversarial experiences of the welfare system greatly compounded this threat, considerably impacting mental health. The stigma of receiving support and dissatisfaction with welfare systems fed into a reluctance to seek financial assistance among those experiencing short-term unemployment, as they weighed up the benefits of financial support with the threat to their self-worth. It may be that this reluctance is no longer feasible when unemployment becomes long-term. Given that financial resources were often needed to engage in other known coping strategies (as has been noted elsewhere) [23], this reluctance can further diminish coping capacity, which may in turn negatively impact readiness to re-enter the workforce and capacity to succeed upon doing so. Quality of life while relying on welfare payments was generally described as suboptimal, which is known to be the case in Australia [58–60], and even though payments initially increased during the COVID-19 pandemic which had the capacity to improve mental health, these measures were short-lived [59].

While job-seeking was perhaps the clearest example of problem-focused, active coping, the process itself negatively impacted mental health, further complicating coping efforts. This echoes the negative associations found in the literature between job-seeking and mental health or well-being [2, 24, 25]. Investing in job-seeking success as the core marker of self-worth during unemployment is problematic given the low success rate of any particular job application, meaning that rejection of a job application is interpreted as a rejection of one’s value, decreasing motivation to pursue further opportunities for rejection. Therefore, balancing job-seeking with other meaningful activities appears crucial to maintaining well-being, and ultimately job-seeking motivation during this time [33]. Employment services and welfare systems need to acknowledge that job-seeking may not always be a viable means to cope immediately following job loss, however, as the difficult job loss experiences among the women in our sample significantly impacted mental health and required time to recover from. Furthermore, re-employment may begin to ease financial burdens, but should not be assumed to entirely resolve the mental health issues faced during unemployment. While quantitative research has found aggregate improvements in mental health upon returning to work [1, 14], more modest improvements are found for those entering into less stable forms of employment [61], and the current findings suggest that mental health issues are not necessarily resolved immediately or for all people. Tailored support to prepare people for a transition back into employment, and ongoing support upon return to work may be necessary for the mental health of some individuals.

The current findings suggest that social connection was only experienced as helpful when it was empathetic to the individual’s circumstances, did not increase job-seeking pressures, or helped to normalise unemployment. This contrasts against common reports of an overall positive association between social support and mental health during unemployment [2, 8, 33]. Within a marginalised population prone to stigma like those experiencing unemployment, the quality rather than the quantity of social support [62, 63] appears particularly critical and consequential for mental health. When striving for connection and support, interactions that reinforced negative assumptions or did not fully appreciate participants’ own frame of reference, left them feeling as though their needs, values, or goals were ignored or invalidated. This, in turn, increased the tension and disconnection between their own and others’ understanding of themselves and their situation, worsening their sense of isolation. It should be noted that both anticipated and encountered judgment from others (experienced during short-term and long-term unemployment, respectively) could negatively impact participants’ mental health. Thus, recommending social support as a beneficial coping strategy during unemployment should be approached carefully by service providers, with the nuances of the source and quality of this support requiring consideration. Families, for instance, have been found to be a key source of both beneficial support and detrimental social pressure during unemployment [64]. The COVID-19 pandemic had a complex relationship with social connection, as it impeded the pursuit of social support while also decreasing feelings of isolation through the normalisation of unemployment and mental health struggles during this time. Although the pandemic had an overarching negative impact on mental health and coping, this study argues that some unanticipated positive outcomes may occur simultaneously.

Participants often knew or learnt coping strategies that worked for them, yet struggled to mobilise these strategies for a variety of intertwined reasons that extended beyond the negative cognitions and emotions noted elsewhere [65]. Participants described internal barriers including diminished motivational resources or mental clarity and self-stigma, in addition to external barriers including diminished financial resources, COVID-19 lockdowns and public stigma. These barriers emerged within the context of participants’ wholistic experience. For instance, the diminished motivation which hindered the maintenance of a structured daily routine was underpinned by a broad sense of diminished control, purpose, self-worth, identity and/or demoralising job-loss or job-seeking experiences. Additionally, the intentional social withdrawal that prevented seeking quality social connection was underpinned by financial strain, shame and stigma around not meeting societal expectations, and/or the COVID-19 lockdowns. Thus, it is not enough to promote routine, quality social connection, or other known coping strategies for someone experiencing unemployment, as their broader situation must be taken into account for such strategies to ultimately prove effective. Clinicians and interventions in this space need to go beyond provision of coping skills and strategies and work toward a wholistic approach to the complex network of factors impacting mental health and underlying barriers to coping. With stigma and shame being common barriers to coping, interventions should consider creative solutions to maximise privacy and evade stigma. The emergence and availability of digital mental health tools may be an important means of overcoming these barriers [66, 67].

### Unemployment as a catalyst for new understandings

New understandings and personal growth commonly emerged out of the experience of unemployment, whereby the disruption to individuals’ lives and worldview triggered deliberate meaning-making processes [68] that generally promoted positive coping. This contributes to a growing area of research finding evidence of growth during unemployment using both quantitative [69] and qualitative [37] approaches. Participants’ growth often involved re-evaluating their priorities and re-orienting their stance toward the future, consequently creating new life and career goals to pursue deeply held personal values. This shift reflects an increase in autonomous motivation, which is associated with a broad range of mental health and well-being indicators (including within workplace settings) [70, 71], and among participants appeared to contribute to a reinvigorated sense of clarity and meaning in life. Therefore, viewing unemployment as an opportunity to facilitate such growth may provide a valuable means for improving outcomes both during unemployment and into the future. For instance, by pursuing careers which one is autonomously motivated toward, they are likely to perform better and be more satisfied in their job [71].

Service providers could potentially make use of these growth processes to encourage an expansion of one’s career options that may improve job-seeking efforts and success—provided these options are well-aligned with individuals’ interests and values. It is important to also recognise that for some, these same meaning-making processes can result in disillusionment with sociopolitical systems that may be demotivating and will require additional sensitivity when assisting with job-seeking. At the same time, service providers may encourage the pursuit of valued goals outside of the job-search context, or shifts toward more short-term markers of achievement and success, as means of buffering the negative mental health impact of unemployment. The development of a more compassionate worldview could also be beneficial to fostering an increased sense of community and ameliorate the isolation inherent to the unemployment experience.

### Limitations and future directions

There are several potential limitations of the study. Firstly, participants were recruited from a well-known mental health research institute website, and may therefore represent a biased sample of individuals with high mental health literacy. Nonetheless, the nation-wide reach of the organisation and use of maximum variation sampling helped to capture a broad range of perspectives. Secondly, while subgroups of participants based on gender, age and duration of unemployment were explored for marked differences in thematic content, these analyses were only intended to provide further contextualisation of participants’ experiences (as is a focus of phenomenological approaches), and caution must be taken not to interpret these findings as generalisable group differences. Furthermore, these subgroups are not exhaustive, and future research may consider screening and comparing participants based on other relevant demographics such as diagnosed mental health issues. Thirdly, the current methodological approach is also not well-suited to explore processes of change over time. Research using narrative approaches may therefore bolster the present findings by illuminating how mental health and coping develop over the course of unemployment and exist within a broader life story [72].

Further work will be needed to continue studying how mental health and coping are experienced during unemployment, as the social, political and economic climate continues to shift and adapt in light of the COVID-19 pandemic. Moreover, welfare systems across the globe are continually shifting (e.g., the recent upheaval of the Australian welfare system) [73], and researchers, service providers and policy makers should consider the potential for additional mental health impacts of this socioeconomic uncertainty on those experiencing unemployment. Any mental health prevention or promotion strategies applying the present findings will also need to consider factors inherent to the context in which they are implemented, including welfare systems or attitudes toward work that differ from those in the current Australian context. Although the current study focused on unemployment, underemployment (inadequate working hours) is likely to relate to similar mental health and coping challenges [74]. Extending investigation of mental health and coping to this population is a priority for future research in light of the recent rise in the gig economy and casualisation of the workforce [75].

## Conclusions

The present study contributes to a nuanced understanding of how people struggle with and are able to overcome a range of interrelated mental health and coping challenges experienced during unemployment. It sheds new light on how an individual’s personal and broader sociocultural contexts shape these experiences, particularly around the struggle to reconcile societal and personal views of which work-related contributions are valuable. It also reveals the complex nature of coping strategies used during unemployment, which can have both positive and negative effects, or can be difficult to instigate or maintain. Importantly, this study recognises that despite negative impacts of unemployment, there is also a potential for personal growth during this time. To better meet the mental health needs of those experiencing unemployment and facilitate effective coping, service providers may benefit from (a) balancing the economic and psychosocial functions of work, (b) approaching coping as a wholistic issue that requires addressing intertwined barriers to engaging effective coping strategies, rather than just raising awareness of these strategies, and (c) being aware of, and engaging with, the opportunities for self-development that unemployment can create as a means to promote well-being.

## Supplementary Information

**Additional file 1.** Supplementary Quotes.

## Data Availability

Complete raw data are not publicly available to preserve participants’ anonymity in line with the obtained ethical approval for this study. However, supplementary data supporting the current analyses can be found in Additional File 1, and further de-identified data can be made available upon reasonable request from the corresponding author.
