# Supplementary material for: Exploring the lived experience of mental health and coping during unemployment
Source: BMC Public Health. 2022 Dec 28;22:2451. doi: 10.1186/s12889-022-14858-3 (PMC9797253; doi:10.1186/s12889-022-14858-3)
Supplement: Supplementary file 1 — Additional file 1. Supplementary Quotes. [file 12889_2022_14858_MOESM1_ESM.pdf]

Additional File 1.

Supplementary quotes related to the lived experience of unemployment

Theme	Quote
**Theme 1: Disrupted identity and direction in life**	*"I'm in limbo. Uncertain"* (P03_F_LT_30–44).<br><br>*"...you just feel like there's no purpose, you know. You don't have a job, what's the point? And so I get very depressed."* (P04_F_LT_45–65)<br><br>*"I'm not the kind of person that can go to work every day and do a half-assed job. ...if I'm doing some something I'm doing it properly, and putting in all my effort. And it's almost like, I've done that my whole life and where am I?"* (P08_F_ST_30–44)<br><br>*"…because I didn't have any purpose and… I didn't feel like I had any sort of accountability to do anything, that's when I started feeling more depressed and demotivated and what-not."* (P09_F_ST_18–29)<br><br>*"...it's quite stressful. ...I was, like, in a state of not knowing what to do, a bit confused, and like, constantly trying to think of a solution."* (P15_M_ST_18–29)<br><br>*"...those first, probably six to eight months [after becoming unemployed] that was just like, oh, this is frustrating and messy. And, what am I going to do about money? What am I going to do about staying in this country?"* (P01_F_LT_30–44)<br><br>*"...the grief that you're going through, and the loss, and that mental anguish… and total confusion in what you thought was the world that you lived in."* (P03_F_LT_30–44)<br><br>*"…to get a lump sum payment is great, but then the fear of not being able to work again. And I'm turning fifty in a month as well, so that, sort of, brought things to a head. Like, 'Am I going to be employable after this?'"* (P10_M_ST_45–65)
**Theme 2: Navigating conflicting views of contribution and progress**	*"…society treats you differently when you're attached to a role, or a status, or a perceived profession that gives you --- you know, societal acceptance."* (P03_F_LT_30–44)<br><br>*"When you say [to other people] you're unemployed, it's kind of like [groans and rolls eyes] 'You're unemployed.' But then also I feel it myself, you know, [groans and rolls eyes] 'I'm unemployed.'"* (P04_F_LT_45–65)

	"…interacting online with strangers… and them saying, 'I worked all these horrible jobs.' You know, 'You shouldn't bludge off the taxpayer, blah, blah, blah.'" (P13_M_LT_30–44)<br><br>*"I'm not working so I need to contribute somehow"* (P01_F_LT_30–44)<br><br>*"I'm not even really enjoying being home because I feel like it's wrong. Like, I should be working."* (P08_F_ST_30–44)<br><br>*"you just feel useless."* (P04_F_LT_45–65)<br><br>*"[My parents were] just like, 'Have you thought about doing like payroll, accounting or something?' Things I have no interest in really. They're like, 'You need a job, you need to support yourself.' Like, 'Hey, look, I have this job. And I don't love it, but I don't hate it either. It pays pretty well. Have you considered doing the same thing?' And none of these things were directions I really, like, needed to think about I guess."* (P11_M_ST_18–29)<br><br>*"Even my sister said, 'why don't you just work in the bakery? They're hiring at the moment. You like making cakes.' And I'm like, you're missing the point, right? I don't mind making cakes, I don't think I'd hate working in the bakery. But I can't afford to lose momentum on developing my career, just because I'm unemployed."* (P13_M_LT_30-44)<br><br>*"…I got some temporary work doing a kid's coding camp, an after school or school holiday program. And I was grateful for the little bit of money. It was, though, not a job I really wanted. My background's in education, but it is not in primary aged education… And the feeling was, is this the only work I can get? Like, is this all it's gonna be? It was a challenge."* (P01_F_LT_30–44)
**Theme 3: Knowing how to cope is not enough**	
3.1 Known coping strategies can help	*"I live out of home, I rent by myself. [The JobSeeker payment] wasn't enough, really, to live without financial stress."* (P09_F_ST_18–29)

but also hinder mental health

“…undergoing the Centrelink process, I think that was the hardest. Because you explain your situation to multiple people and not one of those people says, ‘Oh, I'm sorry to hear that.’ It's like, ‘Okay, cool. Where's this document?’” (P06_F_ST_18–29)

“…I'd go to these appointments and explain the situation. I'm like, ‘Look, I've got a master's degree, I'm trying to get my first graduate job.’ And they'd be saying ‘Well, working at Centrelink isn't my dream job. Go suck it up.’ Or something” (P13_M_LT_30–44).

“…when you’re on unemployment benefits, you have to look for a certain amount of jobs a week. It’s a ridiculous amount. So people are applying to jobs they’ve got no chance of getting. And they put in this phone line, that if people didn’t show up for the interview, the employer would ring this hotline, and dob-in this person, and they could stop their benefits. “ (P04_F_LT_45–65)

“In the long term [applying for jobs] became quite stressful, because… it almost felt like ‘this is never gonna happen.’” (P05_M_ST_30–44)

“I have a master's degree, I'm pretty educated, and I think I'm good at my job. So there's a lot of doubt then [when you receive rejections]. You're like, ‘am I actually as good as I think I am if these… entry-level customer service jobs don't want me?’ That's a challenge.” (P01_F_LT_30–44)

“…last year, right when we were in the middle of lockdown, I decided to apply for a job and see what would happen. And there were like 4000 people for one job. And it's just like, ‘Sure.’” (P04_F_LT_45–65)

“you can only spend so many hours writing job applications, despite what the government says. And then you’re just… exhausted.” (P04_F_LT_45–65)

“…communication [with former colleagues] from being all day, every day has gone to --- I don't hear from them at all.” (P08_F_ST_30–44)

“…[work] gives you an instant connection of, ‘you're part of an organization. This is your role within the organization, to achieve X, Y, Z goals.’ That's an instant community and connection there. Whereas I think when that's not there, that can be very isolating” (P11_M_ST_18–29).

	"…one of the best compliments I can give [my partner] is that I never felt external pressure from them to get a job… I think the most pressure they ever put on me was just a check-in. Like a 'How are you going with this?' 'Do you need any help or support from me?'" (P11_M_ST_18–29).<br><br>*"A lot of the time, my husband will tap me on the shoulder and say, 'Hey, you're in a bad headspace at the moment. Maybe you can go play with a stress ball, or look at the [wellbeing] app on your phone, or something.'"* (P14_NB_LT_18–29)<br><br>*"[online mental health pages help] because I can relate to it and you don't feel like you're alone. It makes you realize that there's so many other people that are going through the same thing."* (P08_F_ST_30–44)<br><br>*"the fact that I was made redundant through the COVID period last year, you know that you're not alone."* (P02_M_LT_45–65)
Theme 3.2: Effective coping strategies are known, but challenging to mobilize	*"…you adjust to sitting with uncertainty, and allowing that to be okay."* (P07_F_ST_45–65)<br><br>*"…you'll learn a lot from each of those experiences [of redundancy], and every little bit that you learn… adds to your personality and your character"* (P02_M_LT_45–65)<br><br>*"I've learned – I've grown through it. I'm on a mental health plan. I'm seeing a psych. It's all been growth."* (P10_M_ST_45–65)<br><br>*"…because I wasn't doing much, just the act of applying for one or two jobs made me feel like I was doing something. …I think that made me feel better and gave me some sense of progression."* (P11_M_ST_18–29)<br><br>*"I feel [mindfulness apps are] really good once you've already started to calm down… if you're really struggling, they're not as useful."* (P09_F_ST_18–29)<br><br>*"When I'm in a good stable place …I will try to do volunteer work. And I try to exercise and catch up with friends. …do my hobbies, um, like reading and cooking.…I try to keep up with those things, but again, if my mood drops then I'm like, 'Forget about it.' And I just get super, super depressed."* (P04_F_LT_45–65)

	"When I first became unemployed, I just let myself have some time to feel what I wanted to feel. I was upset, I was angry, I was just everything under the sun. And I said to myself, 'You know what, I'm not going to let this bring me down.' You know, give myself a couple of days to just feel really crap about it. But it's been a couple of months, and I'm still not feeling much better." (P08_F_ST_30–44)<br><br>*"When I'm in that [depressed] state, I don't want to see anyone or talk to anyone."* (P04_F_LT_45–65)<br><br>*"I'm very good at withdrawing when I'm not feeling good. ...my brain goes straight to like, 'You're a burden.' 'They don't want to know about it.' 'Everybody's struggling.' ... Like, 'Get over it.' Which obviously isn't helpful."* (P09_F_ST_18–29)<br><br>*"Talking to friends and stuff helped, obviously. But virtually is hard, it's not as good."* (P09_F_ST_18–29)
**Theme 4: Unemployment as a catalyst for new understandings**	*"...should I give up on this career dream and do something else?"* (P13_M_LT_30–44)<br><br>*"I think there was an aspect of [the job-seeking experience] which was a cynicism about the world in general ...bigger picture stuff. Like, 'What's the point of all this?' 'What's the point of working?' 'Capitalism sucks.' Like, 'Why do I even need to look for a job?'"* (P11_M_ST_18–29)<br><br>*"[working out my values] sort of directed me towards my next - like, I need to get a role now, just to bring money in for six months so [my family] can get back on an even keel. And then once that's done, my next job will be about giving back. Like, it ...won't be pushing more crap out into the universe. It'll be actually giving back."* (P10_M_ST_45–65)<br><br>*"...even if for the rest of the week I achieved nothing, if I contributed in the [volunteer] meeting, or even attended the meeting, then at least I had done something."* (P09_F_ST_18–29)<br><br>*"I expected [unemployment] to be hard, but I didn't expect it to be as hard as it was."* (P09_F_ST_18–29)
